# Utilization of Slaughterhouse Waste in Value-Added Applications: Recent Advances in the Development of Wood Adhesives

**DOI:** 10.3390/polym10020176

**Published:** 2018-02-11

**Authors:** Birendra B. Adhikari, Michael Chae, David C. Bressler

**Affiliations:** Department of Agricultural, Food and Nutritional Science, Faculty of Agricultural, Life & Environmental Sciences, University of Alberta, Edmonton, AB T6G 2P5, Canada; badhikar@ualberta.ca (B.B.A.); mchae@ualberta.ca (M.C.)

**Keywords:** slaughterhouse waste, specified risk materials, hydrolysis, protein recovery, wood adhesive, adhesive strength, water resistance

## Abstract

Globally, slaughterhouses generate large volumes of animal byproducts. While these byproducts are an important resource of industrial protein that could potentially be utilized in various value-added applications, they are currently either underutilized in high-value applications or being used for production of relatively low-value products such as animal feed and pet food. Furthermore, some of the byproducts of animal slaughtering cannot enter food and feed chains and thus their disposal possesses a serious environmental concern. An innovative utilization of the proteinaceous waste generated by slaughterhouses comprises of waste processing to extract proteins, which are then incorporated into industrial processes to produce value-added bio-based products. In this report, we review the current processes for extraction of protein from proteinaceous waste of slaughterhouses, and utilization of the recovered protein in the development of protein-based wood adhesives.

## 1. Introduction

Meeker (2009) defines a byproduct as “a secondary product obtained during the manufacture of a principal commodity”. The author adds that “one third to one half of each animal produced for meat, milk, eggs, and fiber is not consumed by humans” and is considered a byproduct of the livestock and poultry industries [1]. The approximate live weight percentage of different animals that is considered inedible material is as follows: 49% for cattle, 47% for sheep and lambs, 44% for pigs, and 37% for broilers [2,3].

The Food and Agriculture Organization (FAO) of the United Nations estimates that the aggregate meat production of the world, with the exception of China, is on the rise by 1.9% per year [4]. Consistent with this, the amounts of byproducts generated by the meat industry are also on the rise worldwide. Part of the waste materials, mostly offals, are collected and processed by the rendering industry to produce raw materials that are used in animal feed and pet food. Meat and bone meal (MBM), meat meal, poultry meal, hydrolyzed feather meal, blood meal, fish meal, and animal fats are the primary products resulting from the rendering process [3].

The appearance of “mad cow” disease—a neurodegenerative disease believed to be caused by prion proteins—has completely eliminated certain tissues of bovine, as well as caprene animals, from human consumption. These tissues of ruminant animals are collectively termed as specified risk materials (SRM) [5,6,7,8,9]. In certain countries, legislation restricts the use of SRM in animal feed, pet food, and even in fertilizer applications [6,7]. Consequently, substantial amounts of slaughterhouse byproducts are being incinerated or land filled. This leads not only to additional production costs and economic loss to the meat industry, but also has a negative impact on the environment.

Production of pet food and animal feed by processing of animal byproducts through renderers is the predominant route for utilization of animal byproducts generated in slaughterhouses. However, this route typically produces low-value products such as meat and bone meal, blood meal, feather meal, and poultry byproduct meal. In recent years, the average annual price of such rendered products has been declining. For instance, in 2016, the average annual price of porcine meat and bone meal as well as blood meal each decreased by 17%, and that of feather meal decreased by 25% as compared to their prices in 2015 [10]. A market research report published by RENDER magazine revealed that the price of such products significantly decreased in 2015 as compared to 2014 [10]. Furthermore, due to the fears of spreading Bovine Spongiform Encephalopathy (BSE), the use of meat and bone meal in ruminant feed has become questionable. It is therefore necessary to find innovative ways to obtain increased value for slaughterhouse byproducts, and a renewed interest lies in developing technology platforms for utilization of such slaughterhouse waste in value-added industrial applications.

Recent advances on utilization of slaughterhouse byproducts in various applications includes its use as feedstock for anaerobic digestion [11,12,13,14]; as a source of protein hydrolyzate, enzymes and lipids [15]; for production of processed food for human consumption [16]; and for recovery of bioactive peptides [17]. Very recently, we reviewed the potential of obtaining industrial protein from several renewable resources, and recent approaches of developing protein-based adhesive formulations [18]. Despite being a rich source of industrial protein, the proteinaceous waste generated by slaughterhouses is a very challenging raw material to utilize in functional applications because it is non-homogeneous in composition, often mixed with non-proteinaceous materials, and has poor solubility leading to very limited processability [1,19,20,21]. Moreover, slaughterhouse wastes are potentially contaminated by several pathogens [22], with some (such as SRM) requiring special processing conditions to incorporate them into mainstream value-added applications [5,6,21,23,24,25]. Hence, an important step in developing technical applications of such wastes requires finding the suitable processing conditions and protein enriching methods to transform them into more uniform, soluble, easily processable, and safe, protein-enriched feedstock. This report reviews the current processes in protein extraction and recovery from slaughterhouse waste, and their utilization in the development of adhesives for wood bonding applications as an innovative approach to increase the value of slaughterhouse byproducts.

## 2. Protein Extraction and Recovery from Proteinaceous Biomass

Slaughterhouse wastes comprise the inedible tissues/parts of the animals slaughtered for production of meat, as well as blood, fat, bones, and other materials found associated during the processing of slaughtered animal. Protein recovery from inedible tissues is an essential step in valorization of slaughterhouse waste, but proteinaceous tissues generally have poor solubility in water. Hence, a general practice for protein extraction from such wastes requires solubilizing the protein in an aqueous medium with the aid of heat, chemicals, and enzymes (or combinations thereof), followed by work up of the hydrolyzate to recover partially hydrolyzed protein.

**Figure 1:**
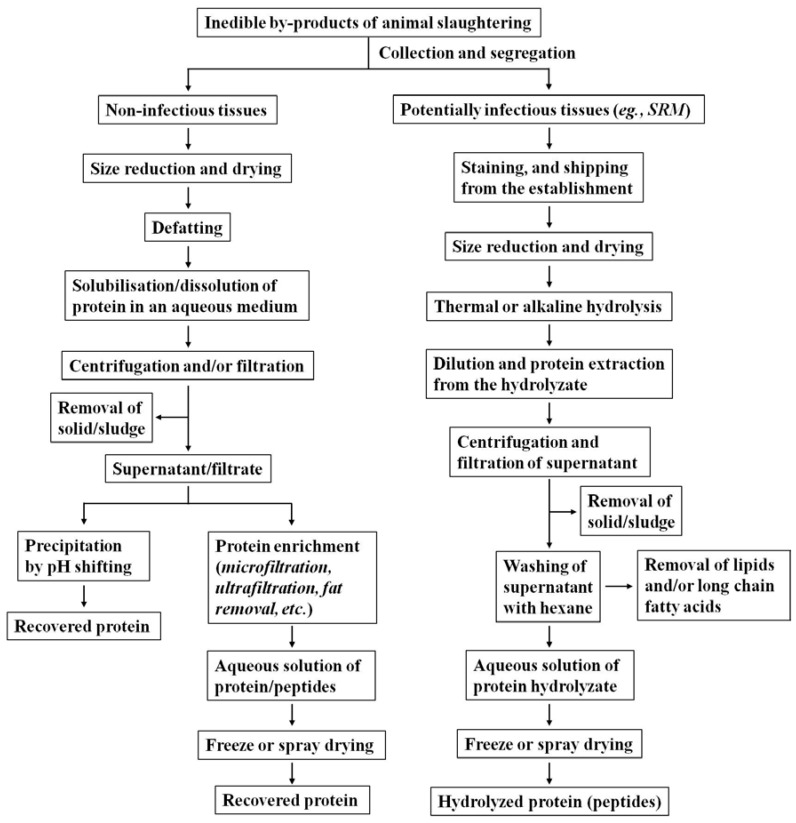
Process flow chart for recovery of protein and/or hydrolyzed protein fragments from slaughterhouse waste.

As slaughterhouse waste is likely contaminated with several pathogens, as well as prion proteins, that are capable of infecting both animals and humans, there are potential risks of infectivity associated with certain tissues [22]. Accordingly, segregation and staining of potentially infectious tissues, and deactivation of pathogens and/or prion proteins is necessary to incorporate the protein recovered from such tissues into value-added applications. A review by Franke-Whittle and Insam (2013) on different methods of treating slaughterhouse waste and their effects on inactivation of different pathogens and prion proteins indicates that alkaline hydrolysis is one of the most promising methods for treatment of slaughterhouse waste to deactivate pathogens [22]. Thermal or alkaline hydrolysis is also recommended by the Canadian Food Inspection Agency (CFIA) for deactivation of prion proteins [5,6,21]. Depending on the nature/type of tissues it contains, different approaches/methods can be employed for extraction and recovery of protein. A generalized flow chart for handling and processing of slaughterhouse waste, including potentially hazardous and infectious tissues, for protein recovery is shown in Figure 1.

### 2.1. Preparation of Protein Hydrolyzates

A United States Department of Agriculture (USDA) report on manufacturing and testing of animal glues describes protein hydrolysis as a crucial step in the preparation of animal glues [26]. In several studies, partial hydrolysis of proteins has been found to be beneficial for formulating wood adhesives as hydrolysis promotes unfolding of protein molecules thereby exposing reactive functional groups and making them available to interact with the functional groups of the substrate [18]. A review of the available literature indicates that enzymatic treatment [27,28,29,30,31,32,33,34,35,36], acid or alkaline hydrolysis [21,35,36,37,38,39,40,41,42,43,44,45], and thermal hydrolysis using subcritical water [21,46,47,48,49,50] are commonly used methods for preparation of protein hydrolyzates from proteinaceous waste. Bacterial treatment using keratinolytic microorganisms has also been reported to be effective for hydrolysis of keratin protein [51,52]. These techniques are reviewed in the following sections.

#### 2.1.1. Enzymatic Treatment

A technology patented by Eckmayer et al. (1980) describes a method for making useful products from proteinaceous animal waste such as blood, animal parts, and meat scraps; this method employs enzymatic hydrolysis of the proteinaceous substrate to recover protein from the biomass [27]. The proteinaceous material is treated with a protease in an aqueous hydrolysis medium at a pH in which the enzyme displays sufficient activity. Effective hydrolysis of protein from waste animal tissues has been reported using pepsin, papain, neutrase, and alcalase [28,29,30,32,33,34]. Of these, pepsin and papain work in acidic pH regimes, nutrease performs its activity at around neutral pH, and alcalase demonstrates its protein hydrolytic ability in slightly alkaline pH [28,29,30,32,33,34]. Protein recovery from hydrolyzates is dependent on the amount of enzyme used, the type of feedstock, the reaction conditions, and the length of time for which the enzyme is allowed to act on the feedstock [28,29,30,31,33].

Webster et al. (1982) investigated the efficacy of pepsin (at pH 3.0), papain (at pH 5.5), neutrase (at pH 7.0) and alcalase (at pH 8.5) for producing protein hydrolyzates from slaughterhouse waste and/or rendered product [29]. The most effective hydrolysis was achieved with papain at 50 °C for all substrates. These enzymes could solubilize about 45–85% of the protein, resulting in protein hydrolyzates with moderate to high protein content [29].

Waste bovine blood is a slaughterhouse byproduct that has potential for both animal feed and human food due to its high protein concentration and quality. In an approach to recover protein from bovine blood, the blood cells were hydrolyzed with papain. Whereas the degree of hydrolysis was lower at extreme pH conditions (2.5 and 10.5), the enzyme generated a protein concentrate with 75% protein content of the hydrolyzate at a pH of 7.5 [30].

Bhaskar et al. (2007) used a fungal protease for enzymatic hydrolysis of partially de-fatted sheep visceral mass (stomach and intestines). Hydrolysis with an enzyme level of 1% (*w*/*w*) at 43 ± 1 °C and a pH of 7.1 ± 0.2 for 45 min was reportedly the optimum condition for producing protein hydrolyzate, resulting in 34% hydrolysis of the proteinaceous mass [28]. In a subsequent work on enzymatic hydrolysis of visceral wastes of fresh water carp (*C. catla*), Bhaskar et al. (2008) found that alcalase (1.5%, *w*/*w*) was able to solubilize nearly 50% of the proteinaceous mass at 50 °C and a hydrolysis time of 135 min, which were the reported optimum conditions [33].

Mechanical deboning is a process used in the meat industry where meat is separated from the slurry of ground bones and meat through the application of pressure in a mechanical deboner [34]. The ground meat is then used to make processed foods while the mechanically deboned residue is considered a waste material. However, this waste material is also a potential resource of protein. For instance, the protein content of mechanically deboned turkey residue is about 19% [34]. Fonkwye and Singh (1995) recovered a potentially edible protein hydrolyzate from mechanically deboned turkey residue through enzymatic hydrolysis using papain at 60 °C. In a typical run, the ratio of residue to water was 1:2, and 1 g papain was used per 250 g of residue. After a 1 h treatment, 51% of the residue was solubilized [34].

To recover hydrolyzed protein, enzyme treatment is followed by deactivation of the enzyme and work up of the hydrolyzate. For instance, the post hydrolysis work up of Bhaskar et al. (2007) involved inactivation of the enzyme by maintaining the hydrolyzate at 85 ± 2 °C for 5 min followed by centrifugation to collect the protein-rich aqueous fraction. Similarly, at the end of hydrolysis, Fonkwye and Singh (1995) heated the hydrolyzate to about 95 °C for at least 15 min to inactivate the enzyme [34]. Spray drying or freeze drying of the aqueous protein hydrolyzate allows for recovery of hydrolyzed protein fragments in a powdered form. From the hydrolyzate of sheep visceral mass, protein was recovered by spray drying at an overall yield of about 6% (*w*/*w*) from the mass of the waste [28]. From 1 kg of mechanically deboned turkey residue, an average of l13 g of freeze-dried product was obtained, which had a protein content of approximately 78%, which translates to a 46% recovery of proteins from the crude mass [34]. The studies described above demonstrate that enzymatic hydrolysis holds a considerable potential for preparation of protein hydrolyzates under mild hydrolytic conditions, and allows for good protein recovery.

**Key points**

- Enzymatic hydrolysis enables preparation of protein hydrolyzates from waste streams under relatively mild conditions (i.e., temperature and pH) with moderate to good recovery yields.
- The long processing times, the specificity of proteases in certain pH ranges, and the cost of enzymes, however, limit the application of enzymatic hydrolysis.

#### 2.1.2. Acid or Alkaline Treatment

The conventional acid hydrolysis of proteins uses hydrochloric acid, typically at a 6 N concentration [35,36]. Organic acids such as propionic acid, trifluoroacetic acid, and sulfonic acids have also been used for hydrolysis of protein [35]. Sodium or potassium hydroxides are commonly used to accomplish protein hydrolysis under alkaline conditions. However, since alkaline chemicals are known to promote decomposition of the hydrolysis products, traditional protein hydrolysis studies predominantly used acid hydrolysis [36,44]. Nevertheless, in recent years, there has been considerable interest in alkaline treatment of slaughterhouse waste for solubilizing and extracting partially hydrolyzed protein fragments from this proteinaceous feedstock [21,38,39,40,53].

Yang et al. (2006) hydrolyzed mixed porcine and bovine whole blood meal (without removing fibrin and red cells) with 8% NaOH in water at 120 °C for 2 h to obtain water-soluble, low-viscosity protein hydrolyzate. The recovered protein was used in the formulation of an adhesive for wood composites [39]. Similarly, Wang and Wu (2012) utilized alkaline treatment for solubilization and extraction of protein from spent hen. In a typical run, the spent hen meat was subjected to defatting and myofibril separation from heavy connective tissues, and protein from the isolated myofibrillar layer was solubilized by adjusting the pH to 11 [40]. Alkaline treatment was also used for solubilization and extraction of protein from mechanically deboned chicken residues [54]. A typical protein extraction protocol consisted of mixing 1 part (by weight) of the residue with 1.25 parts (by weight) of water, adjusting the pH of the mixture to 10.5 with 20% (*w*/*v*) aqueous sodium hydroxide solution, and then stirring the alkaline slurry for 30 min [54]. Mekonnen et al. (2013) conducted alkaline hydrolysis of SRM using sodium hydroxide at a level of 9% (*w*/*w*) of SRM input and a minimum condition of 150 °C and 400 kPa for 180 min in an enclosed pressure vessel [21]. These conditions were adopted from a CFIA approved protocol to ensure complete destruction of misfolded prion proteins that may potentially be present [21].

Keratin proteins such as feather and wool are difficult to solubilize under mild conditions. Nevertheless, reports describing the potential of acidic or alkaline conditions to affect hydrolysis of keratin protein are also available. For instance, Gousterova et al. (2003) investigated the hydrolysis of sheep wool [53], and Jiang et al. (2008) studied hydrolysis of chicken feathers [38] in alkaline conditions. On hydrolyzing the wool at a wool:aqueous solution ratio of 1:10 (*w*/*v*) at a pH of 12, temperature of 120 °C, and pressure of 2.0 atmospheres for 20 min, 82% of the wool mass was solubilized, and the resulting protein hydrolyzate had a protein content of 90% [53]. Jiang et al. (2008) prepared chicken feather hydrolyzate by cooking the feathers at a temperature of 120 °C for 2 h in an alkaline hydrolysis medium containing 6% sodium hydroxide and 2% sodium bisulfite. In this protocol, sodium hydroxide hydrolyzes the peptide bonds and sodium bisulfite cleaves the intra- and inter-molecular disulfide bonds [38]. From DSC and NMR spectroscopy analyses of the keratin fiber treated with formic acid vapors, Barone et al. (2006) concluded that formic acid vapor can penetrate the feather fiber structure leading to solubilization of feather protein [37].

Alkaline solutions carry out the unfolding of protein molecules through the destruction of higher order structures of protein. This phenomenon exposes the hydrophilic functional groups of protein to the dispersing medium and helps protein disperse in water [18,55]. Specific to protein recovery from slaughterhouse waste, alkaline hydrolysis also has an added benefit of sterilization as this approach can carry out complete destruction of pathogens, including prions. The use of more concentrated alkaline solutions and/or a higher amount of alkali against the feedstock appears to be beneficial for enhancing the protein solubilization as this approach leads to severe cleavage of polypeptide chains producing smaller, water soluble fragments [21]. However, this could be detrimental for certain applications such as in adhesive formulation where the larger molecular weight polymers generally result in enhanced adhesive strength [18,56,57,58,59]. The added benefit of protein solubilization under acidic or alkaline conditions is that the peptides can be recovered by simple precipitation by shifting the pH. For instance, 92% and 89% of Atlantic herring (*Clupea harengus*) muscle proteins were solubilized at pH 2.7 and 10.8, respectively. On adjusting the pH of the hydrolyzate to 5.5%, 96% and 94% recovery yields of peptides were achieved from acidic and alkaline hydrolyzates, respectively [45].

Mokrejs et al. (2010) reported on solubilization of more than 90% of chicken feathers via a two-stage alkaline-enzymatic hydrolysis of this abundant proteinaceous feedstock [31]. In the first stage, a suspension of ground, degreased feathers in an aqueous solution of KOH (0.1–0.3%) mixed in a 1:50 (*w*/*v*) ratio was stirred at 70 °C for 24 h. In the subsequent step, the pH of the resulting mixture was adjusted to 9 and stirred at 50–70 °C for 4–8 h in presence of 1–5% of proteinase [31]. It follows from the report of Mokrejs et al. that the combination of chemical treatment followed by enzymatic treatment is likely to allow high hydrolysis efficiency of keratin under milder temperature and alkaline pH regimes.

**Key points**

- Protein hydrolysis under acidic or alkaline conditions is a fast and relatively inexpensive process, and allows for high recovery of hydrolyzed peptides.
- For applications such as in adhesive development where high purity protein is not necessarily a requirement, protein/peptides from acidic or alkaline hydrolyzate can be recovered by simply adjusting the pH.
- As alkaline hydrolysis can destroy pathogens, this process serves to simultaneously sterilize potentially infectious tissues and solubilize proteins.
- In cases where extensive hydrolysis of proteinaceous material is undesirable, the process of alkaline hydrolysis appears to be difficult to control.

#### 2.1.3. Thermal Treatment in Subcritical Water

Reportedly, the first experiments on the hydrolysis of proteins were accomplished in 1820 using the acid hydrolytic method [36]. Chemical treatment, especially alkaline hydrolysis, is rapid but often leads to the generation of secondary effluents and sometimes a loss of protein due to the formation of undesirable products [48]. Further, alkaline hydrolysis under harsh conditions leads to severe cleavage of protein producing smaller fragments, which might limit certain industrial applications of recovered protein hydrolyzate. Enzymatic treatment can be carried out under mild conditions, but it is not cost effective as it usually requires expensive enzymes and long incubation times [48]. Conversely, thermal hydrolysis of protein in sub-critical water is emerging as a highly effective and environmentally benign method for producing protein hydrolyzate. Use of sub-critical water hydrolysis for producing amino acids, reducing sugars, bio-oil and gas fuels from biomass wastes has been recently reviewed, and the method has been deemed renewable, sustainable, efficient, and environmentally safe for recovery of useful chemicals/products from biomass [50]. The main drawback of thermal hydrolysis is that the process requires complex equipment and expensive infrastructure.

To isolate essential amino acids and useful functional products from squid muscle, Asaduzzaman and Chung (2015) conducted thermohydrolysis of dried proteinaceous mass using subcritical water in the temperature range of 160 to 280 °C and a pressure of 6 to 66 bar. Reportedly, enhancement in protein hydrolysis was observed with an increase in temperature and pressure, and a nearly quantitative yield of hydrolysis was achieved at a temperature of 280 °C [46]. The likely reason for this is the effect of temperature and pressure on activation energy of the reaction. As proposed by Serewatthanawut et al., the decrease in the density of water at elevated temperatures, the increase in the water dissociation constant (*K*_w_), and the increase of the dielectric constant of water could also account for the increased hydrolysis of the protein at elevated temperature [48].

Asaduzzaman and Chung (2015) suggested that hydrolysis at a lower temperature range (160 and 190 °C) is favorable for higher protein yield as hydrolysis at higher temperatures led to further hydrolysis of partially hydrolyzed protein molecules to smaller peptides, amino acids, and low molecular weight water soluble organic compounds [46]. Severe hydrolysis at higher temperatures was evident from sodium dodecyl sulphate polyacrylamide gel electrophoresis (SDS-PAGE) analysis of protein hydrolyzate in which increasing hydrolysis temperature resulted in lower molecular weight peptide bands [46]. Under subcritical thermohydrolytic conditions, the intermediate polypeptide chains produced as a result of hydrolysis of proteins undergo further hydrolysis to amino acids. Reportedly, the amino acids are highly labile under subcritical hydrothermal conditions, and undergo further degradation resulting in short chain fatty acids [60,61,62,63]. In agreement with the further degradation of amino acids at higher hydrolysis temperatures, Asaduzzman and Chung found that the amino acid yields also decreased with increasing temperature [46]. However, it is worth mentioning that the effect of temperature and extent of hydrolysis is dependent on the protein feedstock as well as end application of the recovered hydrolyzate. For instance, Watchararuji et al. (2008) found that a temperature range of 200 to 220 °C was suitable for production of protein and amino acid from rice bran and soybean meal through subcritical water hydrolysis [49].

Whilst most studies on thermohydrolysis of protein using subcritical water have been focused on the production of amino acids, Mekonnen et al. [24] and Powel et al. [47] investigated the use of subcritical water for production of intermediate peptides from protein. The former group studied recovery of peptides through subcritical hydrolysis of SRM protein, and the latter group produced peptides from three model proteins: hemoglobin, bovine serum albumin, and β-casein. A comparative analysis of the hydrolyzates of subcritical water hydrolysis and enzymatic hydrolysis of proteins by trypsin further indicated that more than 80% protein sequence coverage was obtained following the subcritical water hydrolysis, which was comparable to that obtained following the enzymatic treatment with tryspin [47].

The results of Mekonnen et al. (2015) on subcritical hydrolysis of SRM conducted at temperatures of 180, 200, 220, 240, and 260 °C indicated that the temperature of hydrolysis affects the yield, molecular size distribution, and composition of the protein hydrolyzate [24]. Analysis of molecular weight distribution and released free amino acids exhibited an incremental hydrolysis with increase in hydrolysis temperature [24], which is in agreement with the results of subcritical water hydrolysis of squid protein [46]. Reportedly, the yield of hydrolyzed protein was lowest for hydrolysis temperatures of 240 and 260 °C, possibly as a result of decomposition of proteins and peptides into low molecular weight organic compounds [24]. The results of Kalambura et al. (2016) on alkaline hydrolysis of brain samples of oxen (which is deemed a potential risk material) are also in agreement with the results of Mekonnen et al. (2015) and Asaduzzaman and Chung (2015); the highest measured protein content being observed for alkaline hydrolysis at 135 °C for 2 h, with the protein content decreasing with increases in temperature and/or length of hydrolysis [43].

These results suggest that it is possible, to some extent, to control the molecular weight distribution of peptides in the hydrolyzate through a proper control of hydrolysis conditions. Since the hydrolysis temperature changed the average molecular weight and molecular size of peptides [24,46], it is likely that certain properties of the protein hydrolyzates, such as affinity towards water as well as thermal and mechanical properties, can be altered by properly selecting the hydrolysis temperature.

**Key points**

- Subcritical water hydrolysis operates without the use of corrosive chemicals (acids or bases) and expensive enzymes, and allows for high recovery of hydrolyzed peptides.
- Under certain conditions, thermal hydrolysis with subcritical water is sufficient to destroy pathogens, and is thus applicable for protein recovery from potentially infectious tissues.
- Several reviews on use of subcritical water for protein solubilization have recommended this technology as a sustainable, efficient, and environmentally benign method.
- Thermal hydrolysis requires complex and dedicated infrastructure and is energy intensive.
- Thermal degradation of hydrolysis products are reported when hydrolysis was conducted at higher temperatures.

### 2.2. Extraction and Recovery of Protein and/or Peptides

Proteins are extracted from the proteinaceous material using ionic water, alkali, or acid extraction depending on the pH at which the protein is soluble [21,41,42]. Extraction can also be facilitated using protein denaturing agents such as urea, thiourea, and sodium dodecyl sulfate (SDS) [19]. Purification or protein enrichment is usually achieved by precipitation of dissolved protein [21,40,41] or by successive membrane separation using microfiltration and/or ultrafiltration [21,30,42]. In the precipitation technique, the pH of the solution is brought to the isoelectric pH of the protein so that isoelectric precipitation of proteins and/or partially hydrolyzed protein fragments occurs. In membrane separation-based techniques, separation and purification occurs by size exclusion wherein the membrane permits the smaller species to pass through it while retaining the larger particles/species in the membrane. Microfiltration is useful for removing the colloidal and suspended particles in the range of 0.1–10 µm, and ultrafiltration is appropriate for removing colloidal or molecular structures ranging between 1 and 100 nm. Ultrafiltration also has the ability to separate soluble macromolecules from other soluble species [30,42,64]. Freeze or spray drying of the aqueous solution obtained after membrane separation produces a protein isolate powder.

Selmane et al. (2008) extracted protein from slaughterhouse byproducts such as pork lungs, beef lungs, and mechanically deboned chicken meat (MDCM) using an aqueous extraction medium whose pH was maintained at 4, 7 or 9 [42]. Protein extraction was achieved by centrifuging the suspension (proteinaceous mass in extraction medium; 20%, *w*/*v*) at 10,000× *g* for 15 min. Whilst only 30% of the protein was extracted under acidic condition, improved protein extraction (nearly 80%) was achieved in alkaline conditions [42]. As the isoelectric points of the main protein fractions of all three byproducts were in a pH range lower than 6 [42], the lower amounts of protein extracted in acidic condition was due to isoelectric precipitation of the protein. Following the extraction, concentration and purification of protein extracted from pork and beef lungs was performed using membrane technologies including successive microfiltration (MF) and ultrafiltration (UF). Unfortunately, the high lipid content in the extract of MDCM caused membrane fouling. Thus, isoelectric precipitation was employed for purification of protein extracted from MDCM. Lipid removal from the isoelectric precipitated protein using a hexane:isopropanol (3:2, *v*/*v*) mixture further enriched the protein content of MDCM extract. The yields of protein recovery were 48%, 53%, and 55%, respectively, for beef lungs, pork lungs and MDCM [42]. Even though the extraction method uses mild conditions and the processes are easy to scale-up, alternative methods to membrane-based purification technologies must be used for commercial viability of this technology for recovery of protein from slaughterhouse byproducts.

Meat and bone meal is a product of rendering industries, produced by rendering of fat from unmarketable tissues of slaughtered animals including cattle, swine, and poultry. Physical distribution analysis of meat and bone meal (MBM) has shown that it is composed of varying proportions of bone and soft tissues [19,20]. On average, the soft tissue fraction constitutes nearly 60% of MBM with the bone comprising nearly 38% [19]. The overall crude protein content of MBM is around 50%; the other major components being 10% fat, 10% calcium, and 5% phosphorous [65]. Hence, a simple defatting process can enrich the crude protein content of MBM. For instance, Park et al. (2000) increased the crude protein content of BMB to 57% after fat removal [41]. MBM protein has a median solubility of 5.35% (solubility ranging from 2.20% to 7.22%) in water at pH 7, which makes extraction difficult under mild conditions [20]. Piazza and Garcia (2010) were able to extract only 8.9 g protein in water from 129 g MBM [66]. However, extractability of MBM protein in aqueous medium can be increased by increasing the ionic concentration. Park et al. (2000) extracted protein from defatted MBM by salt extraction using phosphate buffer (pH 5.3) containing 4% NaCl and 0.05% CaCl_2_ or MgCl_2_. The extracted protein was recovered using the isoelectric precipitation method by adjusting the pH of protein extraction to around 4.0 to 4.5 [41]. Reportedly, increasing the concentration of NaCl from 0% to 4% led to notable enhancement in protein solubility, and the protein extractability of MBM increased from 46% (in absence of NaCl) to 57% (in presence of 4% NaCl). Addition of 0.05% CaCl_2_ or MgCl_2_ to phosphate buffer containing 4% NaCl resulted in further improvement in protein extractability, increasing from 57% (in the absence of divalent ions) to 70% and 74% in presence of calcium and magnesium ions, respectively. Addition of such divalent ions caused salting-in of proteins by further increasing the ionic strength of the extraction medium thereby resulting in increased protein solubility [41]. Through the salt extraction procedure, Park et al. obtained 32 g MBM protein concentrate from 100 g defatted MBM. The results of Garcia and Phillips (2009) are somewhat similar to those of Park et al. (2000) in that aggressive protein solubilizing media, such as aqueous solutions of urea, thiourea, and sodium dodecyl sulfate, were able to solubilize an average of 34% of soft tissue protein and 14% of bone protein from MBM [19]. However, mild extraction reagents such as dilute solutions of EDTA, sodium azide, or sodium chloride, or a dilute solution of protease inhibitor cocktail could only solubilize an average of 14% of soft tissue protein and 4% of bone protein of MBM [19].

Post hydrolysis of SRM, Mekonnen et al. (2013) extracted the hydrolyzed protein fragments from the hydrolyzate by: (i) salt buffer extraction; (ii) acid extraction; (iii) aqueous extraction using water alone; and (iv) a combination of salt buffer extraction and membrane filtration techniques. This approach also allowed them to evaluate the efficiency of different extraction methods [21]. The extraction medium for salt extraction was phosphate buffer (pH 6.5) consisting of 4% NaCl and 0.05% MgCl_2_. The soluble peptides were extracted in the raffinate after centrifuging the mixture of hydrolyzate and the extractant (1:4.5, *w*/*w*). Membrane filtration of the salt-extracted raffinate allowed further purification of hydrolyzed peptides [21]. For acid extraction of peptides from the hydrolyzate, 0.1 N HCl was used as the extraction reagent followed by centrifugation, hexane washing (to remove any lipid), and precipitation of the aqueous fraction at isoelectric pH (pH 4.5). In aqueous extraction, a mixture of hydrolyzate and water (1:4.5, *w*/*w*) was agitated for 30 min, which was followed by centrifugation and vacuum filtration (to remove any insolubles), and then the filtrate was washed with hexane to remove lipids [21]. Reportedly, crude SRM contained nearly 44% protein on a dry weight basis [21]. After hydrolysis and purification, the recovered peptides were more than 90% proteinaceous materials [21]. Among different methods of protein extraction and recovery adopted by Mekonnen et al., aqueous extraction yielded higher amounts of peptides, which were also high in protein content regardless of the medium (aqueous or alkaline) of hydrolysis.

**Key points**

- Addition of salts increases the ionic strength of the extraction medium, and enhances protein solubilization.
- Aqueous solutions of protein denaturing agents such as urea, thiourea, and sodium benzene sulfonate also improve protein solubilization.
- Extraction of peptides using water alone is the most convenient, efficient, and beneficial method of extracting proteins from hydrolyzates produced by thermal or alkaline hydrolysis.
- Membrane filtration and ultrafiltration yield good quality permeate with simultaneous concentration and recovery of soluble proteins, but the process is time consuming, and requires expensive membranes that can be fouled.
- Isoelectric precipitation by pH shifting is a very simple but effective method of protein recovery from aqueous protein hydrolyzates.

### 2.3. Hydrolysis under Alkaline Condition vs. Subcritical Water Hydrolysis

Several reports are available on alkaline hydrolysis or subcritical water hydrolysis of proteinaceous feedstock. However, a report by Mekonnen et al. (2013) is currently the only scientific document that studies the alkaline as well as subcritical water hydrolysis on the same feedstock [21]. They analyzed the recovery yield and protein content by adopting four different extraction methods, as well as the molecular size distribution of the peptides recovered, from SRM hydrolyzates obtained by two different hydrolytic methods. For each of the four extraction methods adopted for protein recovery from hydrolyzates, higher recovery yields and a higher protein content of the recovered peptides were observed using subcritical water hydrolysis rather than alkaline hydrolysis of SRM (Table 1) [21]. As evident from the molecular size distribution of peptides recovered from two different hydrolyzates (Figure 1), hydrolysis under alkaline conditions carried out extensive breakage of amide linkages producing a larger proportion of relatively smaller chain peptides and free amino acids. A plausible reason for the observed lower yield of proteinaceous materials under alkaline hydrolysis could be the severe breakage of amide linkages resulting in increased amounts of free amino acids in the hydrolyzate; further decomposition of the resulting amino acids through a mechanism similar to the deamination of proteins was observed through thermal hydrolysis of bovine serum albumin (BSA) [61,62,63].

**Figure 2:**
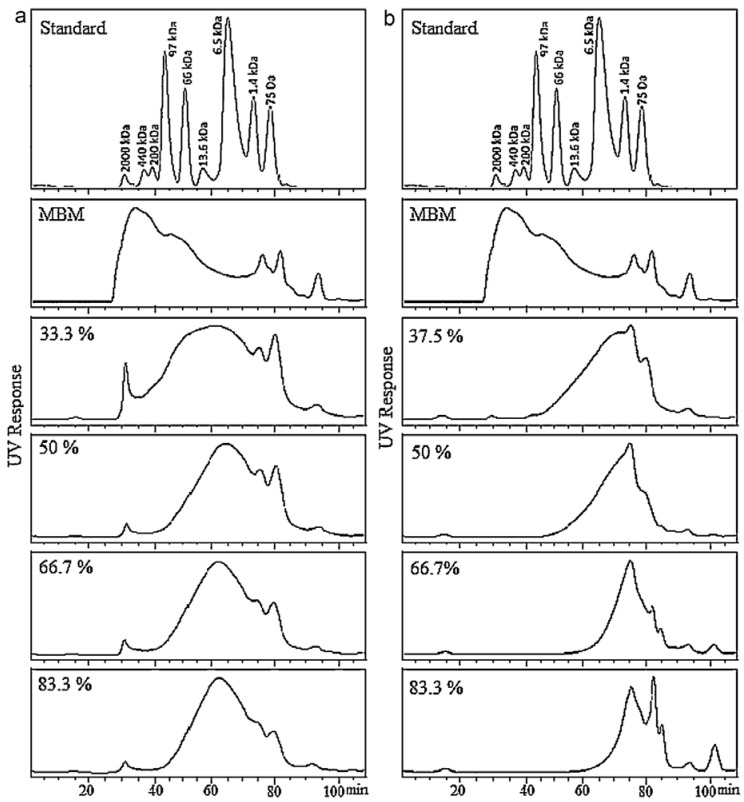
SEC-HPLC of: (**a**) thermal hydrolyzed SRM in 33.33%, 50.00%, 66.67% and 83.33% water, relative to the total mass; and (**b**) alkaline hydrolyzed SRM in 37.50%, 50.00%, 66.67% and 83.33% alkaline solution (15%, *w*/*v*, NaOH), relative to the total mass. Figure adopted with copyright permission from Elsevier (for Mekonnen et al., 2013).

Mekonnen et al. (2013) also studied the effect of the ratio of hydrolysis medium and feedstock on SRM hydrolysis for both hydrolytic conditions. Under both hydrolytic conditions, an enhancement on the degree of protein hydrolysis and a narrower distribution of molecular weights of the recovered peptides were observed on increasing the amount of hydrolysis medium (Figure 2) [21]. Further, for a given ratio of feedstock and hydrolytic medium, the alkaline protocol produced smaller protein fragments and a more homogenous proteinaceous product as compared to the subcritical hydrolysis using water alone (Figure 2). A plausible reason is that alkali catalyzes the breakdown of peptide bonds and facilitates intense hydrolysis of proteins to short chain peptides and amino acids; this was also evident from the fact that the alkaline protocol produced a greater amount of free amino acids than thermal hydrolysis due to the severe cleavage of proteins through such hydrolysis [21].

## 3. Protein-Based Adhesives in Wood Adhesion

Pizzi describes mechanical interlocking, diffusion, electronic interactions, adsorption/specific adhesion, and covalent chemical bonding as the five major phenomena of adhesion [67]. Often, each phenomenon contributes to the overall force of adhesion. In bonding of wood, the principal mechanism of adhesion is believed to involve the interfacial secondary interactions consisting of intermolecular as well as interatomic forces of attraction such as Van der Waal’s forces, dipolar interactions, and H-bonding [56,67]. Pizzi argues, particularly for wood adhesion, that contribution from the phenomenon of diffusion is insignificant, and there is no experimental evidence for the existence of electronic interactions in wood adhesion [67]. Even though some researchers doubt the existence of pure covalent bonds in wood adhesion arguing that there is no direct evidence for the formation of covalent linkages [56,67], there are reports which attribute the adhesive strength of protein-based adhesive systems to the formation of covalent linkages due to the interaction of functional groups of the adhesive formulation with that of wood [23,58,68,69]. Arguably, formation of covalent linkages in wood adhesion seems plausible with some adhesives, especially when the adhesive formulations contain functional groups such as isocyanates [23,58] and azetidinium groups [68,69] that are highly susceptible to react with the functional groups of the components of wood under curing conditions. In fact, azetidinium groups have been shown to react with active hydrogen containing functional groups through the formation of covalent bonds [70,71,72]. Furthermore, as wood is a porous substrate, what is generally accepted in wood adhesion is that a certain amount of adhesive penetration from the wood surface results in effective mechanical interlocking, which is highly desirable and results in stronger bonding [56,67]. Consequently, under specific curing conditions, the combination of any of the chemical interactions and mechanical interlocking may contribute to the overall bond strength in wood adhesion with protein-based adhesives.

In its native form, protein molecules are highly folded, with some of the polar functional groups that can potentially interact with the functional groups of wood being buried inside the folded structure. A recent review by Adhikari et al. (2017) describes protein denaturation or unfolding as an essential step in developing proteinaceous wood adhesives [18]. Denaturation or unfolding of protein by breaking the internal H-bonds of the coiled protein molecules exposes the polar as well as nonpolar structures and reactive functional groups making them readily available to interact with the substrate [18,55,73,74]. Protein denaturation using chemicals or hydrolysis has generally resulted in adhesive formulations with improved dry shear strengths, but such treatments were not successful in achieving sufficient water resistance. Hence, recent studies on the development of protein-based adhesives are focused on chemical modification and chemical crosslinking of protein and/or peptide molecules. Analysis of available literature on protein-based wood adhesives suggests that the most important parameters that determine the strength of an adhesive system are: (i) its ability to create effective mechanical interlocking by penetrating beyond the wood surface; (ii) availability of specific functional groups of the adhesive formulation to result in maximum interfacial interaction; and (iii) cohesive strength within the bulk adhesive, which can be imparted by co-reaction or crosslinking [18,56,58,68,75,76,77]. Additionally, Frihart and Lorenz (2018) suggest that the protein adhesive property is influenced by colloidal property and aggregation state of proteins, and understanding of the structure-property relationship of colloidal clusters of protein will help better understand the adhesive property of protein-based adhesive systems [77].

In the case of urea-formaldehyde (UF) and melamine-urea-formaldehyde (MUF) resins, ageing leads to formation of colloidal particles and their aggregation. The influence of colloidal property of aged UF and MUF resins on wood adhesion has been studied [78,79,80,81,82], which might be relevant to relate the effect of colloidal nature of protein adhesive systems on wood adhesion as well. Pizzi and co-workers found that disruption of colloidal clusters of MUF resin with the aid of an additive results in reduced viscosity as well as surface tension of the resin and imparts better performance in hardening, thereby resulting in improved strength of bonded wood composites [78,79,80]. However, Ferra et al. (2010) suggest that the dispersed phase of colloidal particles is appropriate for strengthening of the adhesive bond, as it acts as a reactive and reinforcing filler at the wood joint surface contributing to the cohesive strength of the resin [82]. This was also evident from the fact that the UF resin adhesive consisting only of lower molecular weight fragments (obtained by removing the colloidal particles) demonstrated poor performance wherein the tested wood specimens mostly underwent failure at the adhesive joint due to lack of cohesive strength [82]. A comparison of particle size distribution and polydispersity range of colloidal aggregates reported in these studies might add significant insight in the influence of colloidal aggregates of the adhesive system in wood adhesion.

The concepts of a model proposed by Ferra and co-workers (2010) for bonding of wood with colloidal aggregates-containing UF resin [82] will be helpful in describing protein adhesion. Similar to what Ferra et al. proposed for UF adhesive systems having colloidal properties, the protein dispersions are likely to contain: (i) a dispersed phase of insoluble molecular aggregates swollen by water on which the soluble oligomers/polymers are in partial agglomeration; and (ii) a continuous phase of soluble oligomers/polymers. When the adhesive slurry consisting of dispersed particles on a continuous phase is applied on a porous surface such as wood, the colloidal particles will remain at the surface acting as reactive fillers and providing cohesive strength. The continuous phase, on the other hand, will be absorbed along with water, and the soluble oligomers/polymers penetrate to some degree within the wood structure assisting in mechanical interlocking.

State of the art technologies for developing protein-based wood adhesives that incorporate the proteinaceous material recovered from slaughterhouse waste focus on two major types of adhesive systems: (i) a protein-phenol-formaldehyde adhesive system; and (ii) formaldehyde-free adhesive systems developed by chemical modification and/or chemical crosslinking of hydrolyzed/denatured protein. In protein-phenol-formaldehyde adhesive systems, protein is co-reacted with the constituents of formaldehyde-based resins either by co-polymerization with the pre-polymers of phenol and formaldehyde or by irreversible incorporation in the phenol-formaldehyde network [83,84]. This approach utilizes protein as a renewable feedstock to replace phenol from phenol-formaldehyde resins. In formaldehyde-free proteinaceous adhesive systems, the denatured protein is either chemically modified or chemically crosslinked with protein/peptide crosslinking reagents. The crosslinking reagents used for such purposes are multifunctional compounds possessing functional groups that are highly susceptive to react with the functional groups of protein and/or peptides [18]. In the following sections, the available literature on different approaches for formulating wood adhesive systems utilizing the protein recovered from slaughterhouse waste is reviewed.

## 4. Utilization of Chicken Byproduct Protein in Wood Adhesive Development

### 4.1. Chicken Feathers

Feathers are a waste product from poultry slaughtering that poses a severe disposal problem and environmental concerns. However, feathers are an incredible resource of protein as they contain 91% protein [52]. Due to their high protein content, chicken feathers are utilized in the production of feather meal after hydrolyzing the keratin protein [15,51,52]. Apart from its application as an additive in animal feed, the potential value-added application of chicken feather protein lies in the development of wood adhesives.

A US patent (Patent No. 2399161; 1946) describes a method for producing chicken feather hydrolyzate for making keratin glue for bonding of wood veneers. The method comprises heating chicken body feathers in a solution of 1% NaOH and 0.5% sodium sulfide at 80–90 °C for 2 h to solubilize the protein molecules [85]. The proteinaceous wood adhesive prepared using the recovered feather protein was used for bonding of wood veneers. The resulting plywood panels had strength comparable to that of commercial plywood under dry conditions, but the glue failed to exhibit sufficient water resistance to satisfy the standards of the Bureau of Standards (test CS 45–40; 1940) for moisture resistant plywood [85]. Heating of the chicken feathers in an alkaline solution unfolded protein molecules and exposed polar functional groups, which could interact with the functional groups of wood veneers resulting in appreciable adhesion under dry condition. However, water absorption by the exposed hydrophilic functional groups weakened the interfacial interactions, and the plywood panels failed to exhibit sufficient water resistance.

Recently, Jiang et al. (2008) developed chicken feather protein-based adhesives by mixing alkaline hydrolyzed feather protein with a mixture of phenol and formaldehyde, followed by heating of the ternary mixture at 90 °C for 2.5 h [38]. In the preparation of this resin, the partially hydrolyzed protein fragments are co-polymerized and/or irreversible trapped in the partially condensed polymer network of the phenol-formaldehyde resin. The adhesive formulations were used for production of fiberboard, and the performance was assessed through evaluation of mechanical properties such as bending strength, bending stiffness, internal bonding strength, and percent thickness swell of the resulted fiberboards. The formulation consisting of one part hydrolyzed feather protein and two parts a phenol and formaldehyde mixture (mole ratio = 1:2) at pH 10.5 performed similarly to that of commercial phenol-formaldehyde resins. Reportedly, this formulation resulted in a 30% replacement of phenol with feather protein as compared to commercial phenol-formaldehyde resins [38]. These results indicated that hydrolyzed feather protein co-polymerizes and/or blends effectively with the components of phenol-formaldehyde resin, and that chicken feather protein has potential as a cost-effective supplement in the manufacturing of phenol formaldehyde wood adhesive resins.

### 4.2. Spent Hen Protein

**Figure 3:**
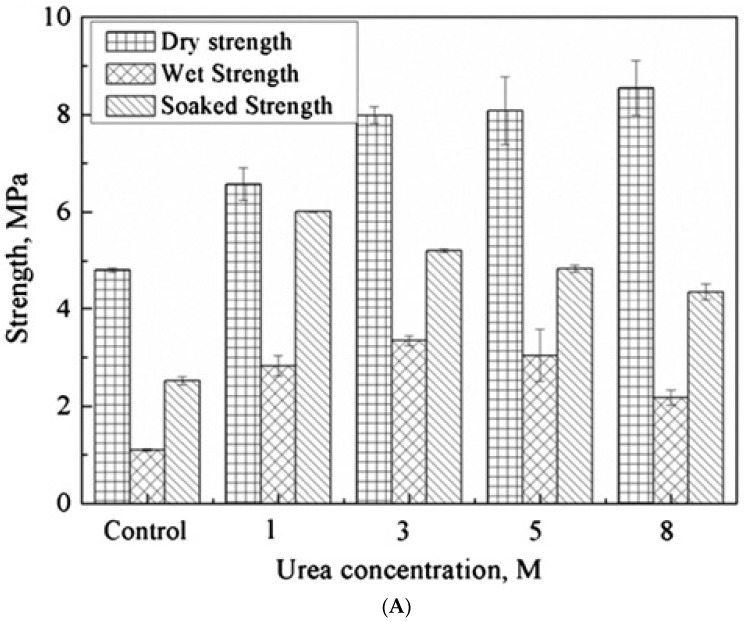

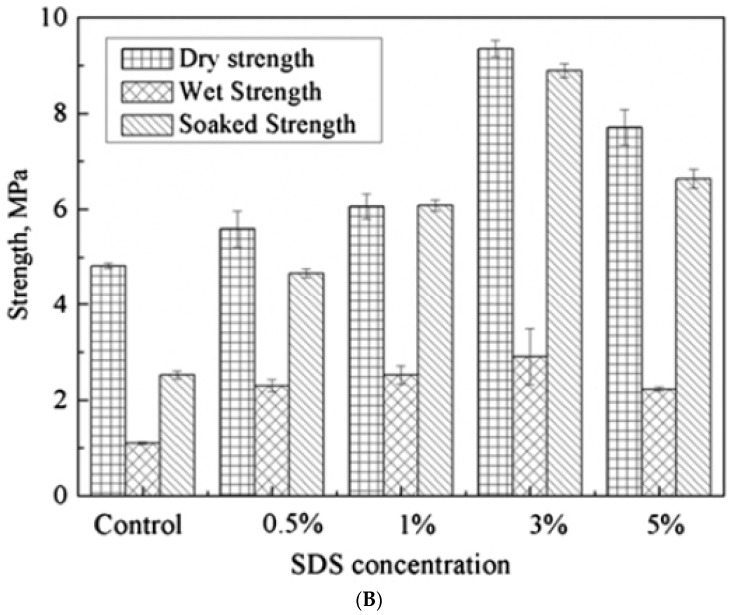
Effect of: urea concentration (**A**); and SDS amount (**B**) on the adhesive strength of modified spent hen protein. Curing conditions: time = 4 min; temperature = 110 °C; pressure = 3.5 MPa. Figure adopted with copyright permission from Elsevier (for Wang and Wu, 2012).

Spent hens are byproducts of the poultry/egg industry and are a potential resource for industrial protein as they contain ~25% crude protein by dry weight [40]. Spent hens are commonly used to produce mechanically deboned meat (MDM), which comprises nearly 20% of the live weight of the bird. The remainder is processed through renderers to produce animal feed additives [86]. Wang and Wu (2012) used spent hen protein to develop wood adhesives after chemically treating the recovered protein with protein denaturing agents—urea and sodium dodecyl sulfate (SDS) [40]. To determine the concentrations of these denaturing agents that were best suited for adhesive preparation, the investigators varied the concentration of urea from 1 to 8 M and the amount of SDS from 0.5% to 5% of protein weight, and evaluated the adhesive strength of resulting formulations (Figure 3).

The authors claim that the dry strength of the adhesives resulting from treatment of protein with urea increased with increasing concentrations of urea up to 8 M, but the formulation developed using 3 M urea exhibited the best water resistance (Figure 3A). However, after review of the data, it appears that the dry shear strength of urea treated protein increased for up to 3 M concentration, and then leveled off on further addition of urea, although a definitive cannot be drawn as statistical analysis was not performed. Whilst the formulation developed by treating protein with 1 M urea demonstrated the best soaked shear strength, the wet shear strength was highest for the 3 M urea (and perhaps the 5 M) treated formulation. In the case of SDS treated protein, the 3% SDS formulation demonstrated the best performance under dry and soaked conditions (Figure 3B). The wet shear strength was also best at 3% SDS, though the wet strength of this formulation is perhaps statistically similar to that of the 0.5% and 1% SDS treated formulation.

Protein-based adhesive formulations containing partially folded structures generally possess higher adhesive strength and water resistance [40]. A plausible explanation is that the intermolecular interactions among polymer chains are stronger when secondary structures remain intact. Furthermore, unfolding of protein molecules increases the surface contact wherein the exposed functional groups of the partially folded proteins interact with the functional groups of wood through interfacial secondary interactions, resulting in improved adhesive strength. Wang and Wu suggest that partial destruction of the tertiary protein structure occurs at lower treatment levels of denaturing agents keeping the secondary structure of protein intact, which results in enhanced adhesion and water resistance. Conversely, higher amounts of denaturing agents are likely to denature the protein such that protein molecules lose their residual secondary structure, resulting in highly disordered polypeptide chains with greater exposure of polar functional groups. Although exposure of polar functional groups can improve adhesive strength by enhancing the interfacial interactions between the adhesive and substrate, over exposure of polar groups likely attracts water resulting in poor water resistance. A balance between the degree of protein disruption and exposure of polar and non-polar functional groups results in optimum bonding strength and water resistance [55,73,74]. For spent hen protein, denaturation with 3 M urea or 3% SDS seems to provide optimal protein disruption for preparing adhesive formulations. Interestingly, these protein denaturing agents had similar effects on the adhesive performance of soy protein [73,74].

With SDS, protein unfolding occurs due to the interactions of the hydrophobic tail of SDS with the hydrophobic side chains of the protein, wherein the polar head of SDS displays electrostatic repulsion with the anionic groups of the protein [73]. Wang and Wu propose that the reduced water resistance at high SDS concentrations was due to the formation of a micelle-like region where hydrophobic groups are buried inside the micelle, thus reducing hydrophobicity of the resulting formulation [40]. However, it is also possible that higher amounts of SDS destroy the secondary structures of proteins and by extension the water resistance property of the final adhesive.

In a very recent US patent (Patent No. US9522515 B2), Wu and Wang (2016) have elaborated the embodiments of wood adhesive preparation from spent hen protein and its application for the preparation of wood specimens from birch veneer [87]. The patent further reveals that the wood adhesive prepared from denatured hen exhibited better strength and water resistance as compared to denatured soy and canola proteins [87].

**Key points**

- Wood adhesive developed from hydrolyzed protein recovered from the waste from the poultry industry have demonstrated appreciable adhesive strength under dry conditions. However, the formulations developed from hydrolyzed protein alone failed to satisfy the required water resistance.
- Adhesive strength and water resistance of chicken byproduct protein-based adhesives can be enhanced through partial destruction of tertiary protein structures with denaturing agents such as urea and sodium dodecyl sulfate. However, the water resistance of adhesive formulations developed from denatured protein alone is still far from being competitive with that of commercial wood adhesive resins.
- To find commercial applications for peptides recovered from the poultry industry in wood adhesive formulations, it is necessary to enhance the water resistance of such formulations. Chemical crosslinking of denatured/hydrolyzed protein or blending peptides with commercial resins are potential ways to address this issue, and the review of the literature indicates that there has been growing interest in this space.
- In protein-phenol-formaldehyde adhesive systems, the protein component replaces appreciable amount of phenol from conventional phenol formaldehyde resins. One such formulation developed by utilizing hydrolyzed protein recovered from the waste of the poultry industry has shown comparable performance to that of phenol formaldehyde resin-based wood adhesive. This demonstrates that the hydrolyzed protein recovered from waste streams possesses tremendous potential in the development of wood adhesives.

## 5. Utilization of Cattle Byproduct Protein in Wood Adhesive Development

### 5.1. Meat and Bone Meal

The potential application of meat and bone meal protein in preparation of bioplastics [88] and flocculating agents [66] has been investigated. However, a report by Park et al. (2000) [41] and a patent by Yang and Yang (2010) are the only scientific documents that describe the application of meat and bone meal protein in the development of adhesive formulations for wood bonding applications. Park et al. developed the adhesive formulations by heating suspensions of meat and bone meal protein in water for 30 min at temperatures ranging from 60 to 90 °C at pH values from 5.0 to 9.0. Adhesive strength and water resistance were evaluated through determination of the force required to break glued joints [41]. Formulations at pH 6.0 and 7.0 demonstrated better performance than those at lower or higher pH, and the best adhesive performance was observed for the formulation prepared by heating a 20% (*w*/*v*) suspension of the protein at 80 °C at pH 7.0. In the range of pH 6.0–8.0 and in the presence of heat, the authors claim that the protein molecules of meat and bone meal will unfold completely and irreversibly, exposing polar functional groups [41]. Accordingly, the enhanced adhesive strength observed at pH 6.0 and 7.0 is likely attributable to increased secondary interactions between the meat and bone meal protein and the wood surface.

The effects of chemical crosslinking of partially hydrolyzed meat and bone meal protein with glutaraldehyde and glyoxal were also examined by Park et al. (2000). Crosslinking of meat and bone meal protein concentrate with glutaraldehyde increased the adhesive strength by nearly 8%, and enhanced the water resistance of the formulated adhesive by up to three times over the control adhesive developed from protein concentrate but without crosslinking [41]. The addition of crosslinking agent in the formulation helped protein molecules form rigid three-dimensional structures with improved bond strength and water resistivity.

A US patent (Patent No. US 20100258033A1) by Yang and Yang (2010) describes a method for preparing wood adhesives from a variety of proteinaceous materials along with an acidity regulator, a curing agent, a preservative, and a filler. The typical adhesive formulation made using meat and bone meal consisted of 100 g of water, 25 g of meat and bone meal, a mixture of 6.0 g of glutamic acid and 6.0 g of sodium citrate (used as acidity regulator), 6.0 g of *m*-nitrophenol (used as an aromatic compound), a mixture of 1.5 g of 2-dodecen-1-ylsuccinic anhdride and 3.5 g of dodecanedihydrazide (used as a curing agent), 11 g of ethoxyquinoline (used as a preservative), a mixture of 0.5 g of polyethylene wax and 0.5 g of chitosan (used as a modifier), and 7 g of titanium dioxide (used as a filler). The inventors claim that the prepared adhesive is applicable for making engineered wood products including veneer plywoods, blockboards, flakeboards, oriented strand board (OSB), medium as well as high density fiberboards, flooring substrates, and laminated veneer lumbers. However, data showing the strength of resulting engineered wood products were not provided. Nevertheless, appearance of such patented technologies for utilization of protein from waste streams for development of engineered wood products is expected to generate significant interest in this regime [89].

### 5.2. Blood and Blood Meal

Slaughterhouses collect approximately 10–22 kg of blood per head of cattle slaughtered [90]. Blood meal—which consists of 80–90% protein—is a dry and stable product obtained after processing of the blood collected in slaughterhouses [91]. As it is rich in protein, the blood meal is typically used in animal feed for augmenting protein and/or amino acid content. In recent years, part of slaughterhouse blood has been used in production of food products such as sausages, minced meat, and restructured meat products for human consumption [16,17]. Blood as well as soluble grades of blood meal have been used for manufacturing adhesives for centuries, and blood-based adhesives are of great historical importance to the adhesive industry. Until the emergence of synthetic resins, blood-albumin glues were the most important water resistant glues available for the plywood industry [92].

Historically, blood-based glues were sold as a dry powder, which could be mixed with water and chemicals to produce a homogeneous and easily spreadable material with alkaline qualities. The chemicals used for this purpose included sodium hydroxide, lime, sodium silicate, or combination thereof [93,94,95]. As blood is a globular protein consisting of highly folded polypeptide chains, alkaline conditions are necessary for protein unfolding and for attaining an aqueous dispersion with suitable viscosity. In addition to providing an alkaline environment, sodium silicate could act as a curing agent [96]. In conventional blood-based adhesives, sawdust, wood flour or other lignocellulosic materials were added to the formulations, all of which typically function as dry extenders [93]. The purpose of extenders was to lower the cost of the adhesive by reducing the amount of primary binder needed per unit area, and also to improve the void filling properties of the adhesive system. Since protein solutions tend to form stable foams that can impact volumetric measurements, defoaming agents such as terpeneol were traditionally used in wood adhesives to ensure equal loading of the adhesive on the adherends [94]. Addition of casein and lime in the formulation lowered the viscosity of the formulation making it more easily spreadable, and increased the water resistance through formation of water insoluble salts of protein molecules with the added calcium ions [93]. In some cases, protein-rich meals such as peanut meal were also added to improve the life of the formulation [93].

For instance, a US patent (Patent No. 1892486) issued in 1932 describes methods for preparing blood-based glue comprising of blood albumin, protein-rich seed meal, and casein, which at time of application could be mixed with water, hydrated lime, and sodium silicate to produce a glue suitable for making waterproof plywood [93]. Similarly, a US patent (Patent No. 1976436) issued in 1934 describes a process of making an adhesive using blood, alkali (NaOH), lime, and sodium silicate, for production of wood panels through cold pressing [94]. The typical formulation contained 100 parts dried blood, 8 parts NaOH, 7 parts lime, 30 parts sodium silicate, 675–725 parts water, and terpineol as a defoaming agent. This formulation resulted in a superior glue that was used to produce wood panels that were practically waterproof and weather resistant. As a result, these panels could be used for outdoor purposes, which was novel for the time. The inventions reported in this patent indicated that positive alkalinity was essential for the formulations to attain sufficient strength during cold pressing [94]. Generally, it was possible to control the viscosity and improve the working life of glue through a balance of alkali and lime in the formulation [97]. Furthermore, the presence of calcium ions enhanced water resistance of the protein-based adhesives due to the slow conversion of the sodium salt of polypeptide chains to water insoluble calcium salts [97].

In a US patent (Patent No. 2292624) issued in 1942, the inventor describes a method for preparing a wood adhesive from blood meal through the hydrolysis of blood protein by boiling in 20% NaOH for up to 30 min. This strong alkali treatment dissolved the blood protein, which, upon cooling produced a blood-based glue that could be easily spread on wood surfaces, and could be used to produce plywood with strong glue bonds and substantial water resistance [95].

A recent US patent (Patent No. 8092584 B2) issued in 2012 describes the use of fresh animal blood (without dehydrating or de-watering) for development of plywood and particle board adhesives. A typical formulation was prepared by adding ethylenediaminetetraacetic acid (EDTA) (10%) and sodium azide (1%) to the fresh blood, wherein EDTA acts as an anticoagulating agent and sodium azide acts as a preservative [96]. Lime was then added to the anticoagulated and preserved blood, and the pH was adjusted to 9–11 by the addition of NaOH. Afterwards, sodium or potassium silicate was added as a curing agent, which was followed by addition of ammonia to produce the final adhesive [96]. Reportedly, addition of sodium silicate resulted in formation of an insoluble protein network and enhanced the water resistance of the blood-based adhesive [96,98]. The adhesive performed significantly better than polyvinyl acetate resin for bonding of maple wood strips. Under dry conditions, this adhesive also performed similarly to phenol formaldehyde resin for construction of three-ply aspen plywood [96].

Lin and Gunasekharan (2010) found that the rheological properties of blood-based adhesives could be altered by controlling pH or temperature [98]. They then investigated the effect of pH on cow blood-based adhesive formulations by examining the bonding strength of three-ply aspen plywood specimens. The bonding strengths under dry and soaked conditions were independent of the pH of the final adhesive formulation in the range of pH 9.3 to 11.2. The dry shear strengths of plywood specimens bonded with the blood-based adhesives were statistically similar to those of the phenol formaldehyde resins. The wet shear strength of the blood-based adhesive was, however, inferior to that phenol formaldehyde resin, and this likely resulted from the hydrophilic nature of the blood protein hydrolyzate [98]. Nevertheless, these results demonstrate the potential application of cow blood-based adhesives in construction of interior grade wood products.

More recently, Lin and Gunasekharan (2016) blended cow blood-based adhesives with acrylic latex-based adhesives and investigated the bond strength and water resistance of the final adhesive system [99]. A blended adhesive led to a notable enhancement of bond strength and water resistance of the resulting wood composite made from hard maple wood under both dry and soaked conditions. For instance, a formulation consisting of 70% cow blood-based adhesive and 30% acrylic latex-based adhesive exhibited 44% enhancement in dry shear strength and 203% enhancement in soaked shear strength as compared to the blood-based adhesive alone. Unfortunately, the authors did not compare the adhesive performance of the blend with that of latex-based adhesive system alone. However, the formulations consisting of 30% and higher amounts of latex-based adhesive demonstrated adhesive strengths and water resistance property similar to that of phenol formaldehyde resins [99]. As latex-based adhesives have high chemical affinity and can easily interact with the functional groups of proteins through non-covalent interactions, it is probable that grafting of blood protein with latex-based adhesives produced physico-chemical crosslinks that resulted in enhanced cohesive strength of the bulk adhesive system and improved bond strength. Moreover, as reported by the authors, the addition of latex-based adhesives to the blood-based adhesive system led to a decreased viscosity [99], which presumably led to enhanced penetration of the final adhesive, thereby resulting in improved mechanical interlocking and enhanced bonding strength. On the other hand, addition of latex gives plasticity to the bondline of protein-based adhesives that reduces the brittleness of the cured adhesive and helps to retain high bond strength [97].

Yang et al. developed a blood protein-based adhesive resin by vigorously mixing the hydrolyzates of blood meal with partially condensed phenol formaldehyde resin at 50 °C to get adhesive formulations consisting of 50%, 60%, and 70% protein hydrolyzate [39]. These formulations had a solid content of about 42% and a pH of about 9.5. In these adhesive systems, the protein molecules were either co-reacted or irreversibly incorporated into the phenolic resin networks. A formulation containing 70% blood meal hydrolyzate and 30% phenol-formaldehyde resin resulted in medium density fiberboards (prepared by bonding equal parts of juvenile hybrid poplar fiber and cornstalk fiber) that met the requirements of the American National Standard Institute (ANSI) for interior as well as exterior grade fiberboards in modulus of rupture, modulus of elasticity, and internal bond strength, surpassing those of boards bonded with neat phenol formaldehyde resin. However, after 24 h, the thickness swelling of boards bonded with the protein-phenol-formaldehyde formulation was greater than that of the boards bonded with phenol-formaldehyde resin, presumably due to the hydrophilic nature of the protein hydrolyzate that could absorb and retain moisture. Additionally, fiberboards bonded with blood protein-phenol-formaldehyde adhesive formulations were superior to those bonded with soy and peanut flour protein-based formulations as well as urea formaldehyde resin in all tests of dimensional stability and the thickness swelling test [39]. Whilst the fiberboards prepared using soy or peanut protein-based formulations in combination with phenolic resins only met the requirements for interior grade fiberboards, those made with blood meal protein-based formulations met the requirements for exterior grade medium density fiberboards as well. This was most likely due to the higher protein content found in blood meal compared to soy and peanut flour, which was also evident from the fact that the quality of protein-based resins developed from soy and peanut flour could be upgraded by incorporation of blood protein in the formulations [39].

### 5.3. Specified Risk Materials (SRM)

Mekonnen et al. (2015) developed plywood adhesive formulations using hydrolyzed protein fragments recovered from thermal hydrolysis of SRM using subcritical water at 180, 200 and 220 °C, and investigated the effect of various parameters on lap shear strength under dry and wet conditions [25]. The adhesives were formulated by reacting hydrolyzed SRM protein extracts with partially condensed glutaraldehyde-resorcinol resin. The hydrolyzed protein molecules are believed to co-polymerize with and/or incorporate irreversibly in the pre-polymer of glutaraldehyde and resorcinol. Since crosslinking reactions can occur between the terminal amine groups of peptides and the functional groups of glutaraldehyde, it is possible that the protein hydrolyzates are co-polymerized with the partially condensed pre-polymer of resorcinol and glutaraldehyde. Nine different formulations were developed according to the Taguchi experimental design by varying: (i) the molar ratio of glutaraldehyde and resorcinol; (ii) the weight ratio of the glutaraldehyde–resorcinol resin and protein; and (iii) the protein hydrolysis temperature, and the effect of these parameters on adhesive strength and water resistance were evaluated [25]. Eight out of nine formulations resulted in plywood specimens that satisfied the minimum dry shear strength requirement, and three formulations satisfied the soaked shear strength requirement of American Society for Testing and Materials (ASTM) for urea formaldehyde resin-based plywood adhesives [25]. The formulation consisting of 20% (*w*/*w*) hydrolyzed protein (hydrolysis temperature of 220 °C) and 40% (*w*/*w*) glutaraldehyde-resorcinol pre-polymer (mole ratio of resorcinol:glutaraldehyde = 0.5:1) exhibited the highest adhesive strength under dry conditions. Alternatively, the formulation consisting of 20% (*w*/*w*) hydrolyzed protein (hydrolysis temperature of 200 °C) and 25% (*w*/*w*) of resorcinol:glutaraldehyde (1:1 mole ratio) resin demonstrated the best water resistance property [25].

Additionally, the resorcinol-glutaraldehyde-protein hydrolyzate formulation displayed improved adhesion compared with the glutaraldehyde-resorcinol resin alone. As the peptides within the hydrolyzate contain several amine, carboxyl, and hydroxyl groups, it is likely that the addition of hydrolyzed protein to the glutaraldehyde-resorcinol system led to an enhancement in interfacial contact due to secondary interactions with the functional groups of wood thereby resulting in improved adhesion. It is also possible that the addition of peptides to the partially condensed resin of resorcinol and glutaraldehyde resulted in improved cohesive strength of the formulation due to formation of additional crosslinks. Secondary interactions across the interface of the adhesive and the substrate, and mechanical interlocking of the wood components by adhesive molecules are the primary mechanisms involved in bonding of wood with the glutaraldehyde-resorcinol-protein hydrolyzate formulation [25].

What is much less studied in the field of hydrolyzed protein-based wood adhesive systems is how the extent of protein hydrolysis affects adhesive strength and water resistance. Generally, a higher degree of protein hydrolysis generates smaller protein fragments and a greater number of polar functional groups, which could potentially interact with the functional groups of wood leading to improved adhesion. However, an increase in polar functional groups may lead to enhanced water absorption, which ultimately weakens the interfacial interactions (especially hydrogen bonding) thereby leading to weak adhesion. Mekonnen et al. (2015) came to this conclusion when analysing the effect of protein hydrolysis temperature on the adhesive strength of hydrolyzed SRM protein-based plywood adhesives. Under dry conditions, plywood specimens bonded with a formulation consisting of hydrolyzed SRM peptides hydrolyzed at 220 °C demonstrated the highest lap shear strength. For water resistance, however, the formulations comprised of peptides hydrolyzed at 200 °C displayed the highest soaked shear strength [25]. Mekonnen et al. also noted that formulations containing peptides from hydrolyzates generated at higher temperatures had lower viscosity, allowing for greater penetration of the wood surface and improved adhesion [25].

In pursuit of potential value-added utilization of SRM, Mekonnen et al. developed an SRM protein-based adhesive system for the preparation of oriented strand board (OSB) panels by chemically crosslinking SRM hydrolyzates with 4,4-diphenylmethane diisocyanate [23]. Their adhesive formulations incorporated 40–85% (*w*/*w*; dry basis) hydrolyzed protein and the performance of the resulting OSB panels was assessed by evaluating their static bending strength, internal bond strength, and bond durability according to the guidelines of ASTM (D1037-12) as well as the Canadian Standards Association (0437.0–93). The panels produced using adhesive formulations with 40%, 50% and 60% (*w*/*w*) hydrolyzed protein had static bending (modulus of elasticity and modulus of rupture) values that satisfied the Canadian Standards Association (CSA) requirement. With regards to the internal bond strength, formulations consisting of 40% and 50% protein hydrolyzates satisfied the CSA requirement [23]. In addition to interactions between the functional groups of the hydrolyzed protein and wood, the residual functional groups of 4,4-diphenylmethane diisocyanate could potentially form urethane linkages with the hydroxyl groups of the wood during the curing step, which could also improve adhesive strength. Nevertheless, the principal mechanism of adhesion appears to be the interfacial secondary interactions (such as hydrogen bonding), which is evident from the fact that the adhesive forces were significantly weakened by boiling water; these formulations failed the two-hour boiling test [23].

**Key points**

- Blood protein-based adhesives developed from water soluble blood meal have long been known to produce waterproof composite wood panels, though they lost their market share after the arrival of synthetic adhesives. Nevertheless, the appearance of several reports and patents in recent years suggests that there is growing interest in utilization of blood and blood meal in the development of proteinaceous adhesives.
- Some adhesive formulations developed from fresh blood have shown adhesive strength and water resistance comparable to that of commercial resins used for production of composite wood products.
- Blending and/or co-reacting of blood protein with acrylic latex-based glues as well as with partially condensed phenol formaldehyde resin has resulted in blood protein-based formulations consisting of as high as 70% (*w*/*w*) blood protein that demonstrated adhesive performance comparable to that of phenol formaldehyde resin-based wood adhesives.
- Under identical conditions of adhesive preparation and testing, the blood meal-based adhesives have demonstrated much better performance than those developed from soy meal.
- Thermal or alkaline hydrolysis produces hydrolyzates with low molecular weight protein/peptide fragments, which generally demonstrate poor performance as wood adhesives. However, the use of suitable crosslinking agents has enabled formulation of hydrolyzed peptides-based adhesive systems that satisfy the minimum strength requirements of ASTM for plywood adhesives.

## 6. Chemical Crosslinking: A Key in Enhancing Adhesive Strength and Water Resistance

Studies on the adhesive strength of polymers have demonstrated that a balance of cohesive and adhesive strengths results in stronger adhesion, and the most favorable combination of adhesive and cohesive forces is achieved at an optimum molecular weight of the polymers. Further, the interplay between the molecular weight of polymers and their adhesive strength is governed by the extent of physicochemical interactions and/or crosslinking of polymer chains [57,100,101,102,103]. Additionally, chemical crosslinking generates rigid three-dimensional networks of polymers, and enhances the water resistance property. Under sub-optimal crosslinking conditions, the formulation lacks cohesive strength, which translates to lower bonding strength and water resistance [103]. This section summarizes the effects of chemical crosslinking on adhesive strength and water resistance of adhesive formulations developed from hydrolyzed protein/peptides.

**Figure 4:**
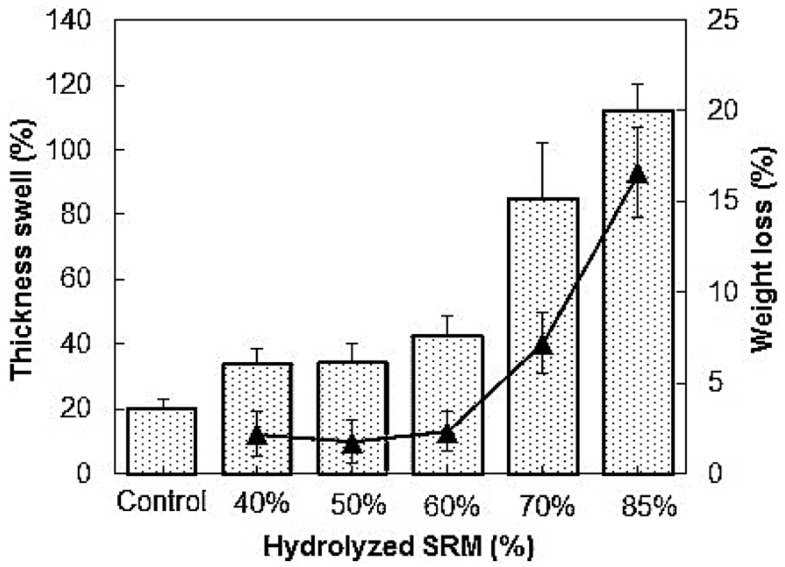
Percentage thickness swell of OSB panels and weight loss of adhesive as a function of the percentage of hydrolyzed SRM in the adhesive formulation. The bar graph shows the thickness swell of OSB panels using hydrolyzed protein adhesives cured with 4,4-diphenylmethane diisocyanate. The scattered line chart shows the weight loss of the cured adhesive in water as the hydrolyzed protein concentration was varied from 0% to 85%. Figure adopted with copyright permission from Wiley-VCH (for Mekonnen et al., 2014).

From the analyses of mechanical properties and water resistance of OSB panels developed from hydrolyzed peptides and 4,4-diphenylmethane diisocyanate, Mekonnen et al. (2014) demonstrated the importance of effective crosslinking of peptides for adhesive production [23]. The SRM peptides-based formulations consisting of 70% and 85% (*w*/*w*) of protein hydrolyzate did not produce OSB panels that satisfied the minimum mechanical requirements of ASTM and CSA [23]. The underperformance of formulations consisting of higher amounts of hydrolyzed peptides was presumably because the peptides were not fully crosslinked, and thus the resulting adhesive system lacked sufficient cohesive strength [23]. Further, any peptides that do not achieve crosslinking are leached out when exposed to water, and this translates to a significant reduction in bond strength. This was evident from thickness swelling of OSB panels and weight loss of adhesive examined by Mekonnen et al. (Figure 4) where the OSB panels bonded with formulations consisting of 70% and 85% (*w*/*w*) peptides exhibited marked thickness swelling due to water absorption and weight loss of the adhesive due to the leaching of non-crosslinked peptides during soaking [23].

**Figure 5:**
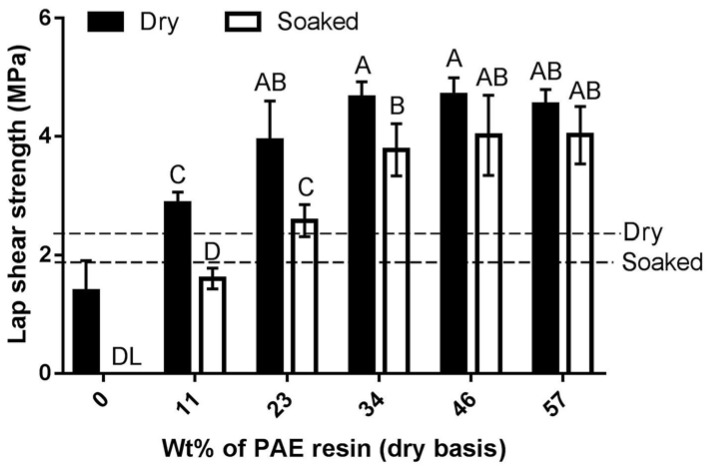
Effect of the weight ratio of peptides and PAE resin on lap shear strength of plywood specimens bonded with the peptides-PAE adhesive. The components of the adhesive system were reacted for 120 min, and the plywood specimens were hot pressed at 120 °C and 3.5 MPa for five minutes. Error bars are the standard deviation of six plywood specimen measurements. Specimens that delaminated when soaked in water are indicated (DL). Means that do not share a letter are significantly different (Tukey, 95% confidence level). The minimum shear strength requirements as specified by ASTM D4690 are shown: 2.344 MPa for dry shear strength; 1.93 M. Figure adopted with copyright permission from MDPI (for Adhikari et al., 2016).

The importance of effective crosslinking of hydrolyzed peptides on adhesive strength and water resistance of the formulated adhesive has also been demonstrated by Adhikari et al. through the evaluation of lap shear strengths of plywood specimens bonded with SRM-peptides and polyamidoamine epichlorohydrin (PAE) resin [75,76]. In the absence of crosslinking agent (i.e., PAE resin), SRM peptides demonstrated weak adhesion (below ASTM requirements) under dry conditions, with virtually no water resistance (Figure 5). The adhesive strength and water resistance increased progressively with increasing amounts of PAE resin in the formulation. Formulations containing 23–57% (*w*/*w*; dry basis) PAE resin displayed adhesive strength and water resistance that satisfied the minimum ASTM requirements [75]. Through the analysis of functional groups of the peptides and crosslinking agent, Adhikari et al. also demonstrated that effective crosslinking was achieved when the molar ratio of reactive functional groups of crosslinking agent and peptides reached 1:1, and further addition of crosslinking agent did not cause notable changes in adhesive strength of the peptides-PAE resin based formulations [75].

Li et al. (2017) recently demonstrated the importance of chemical crosslinking on water resistance of blood meal-based adhesives [104]. Their adhesive formulation consisted of 39 g of blood meal dispersed in 100 g of aqueous solution consisting of 2% (*w*/*w*) polyvinyl alcohol and 1% (*w*/*w*) sodium dodecyl sulfate, and 8 g of triglycidylamine. In this formulation, triglycidylamine is the crosslinking agent, the polyvinyl alcohol is an emulsifier (that prevents the blood meal protein molecules from agglomerating), and sodium dodecyl sulfate is a denaturing agent. The plywood specimens bonded with crosslinked adhesive had average wet shear strength of 1.27 MPa, which represented a 388% enhancement from that of the formulation consisting of blood meal alone (0.26 MPa), and more than 80% higher than the requirement (≥0.7 MPa) of the China National Standard GB/T 17657 (2013) for type II plywood [104]. In contrast, the adhesive developed in a similar manner using soy meal protein resulted in plywood panels that had an average wet shear strength of only 0.84 MPa [104]. The improved performance of blood meal protein-based adhesive is likely attributable to the higher protein content in blood meal compared to soy meal.

The significance of chemical crosslinking of proteins and/or peptides on adhesive strength has also been demonstrated for proteinaceous wood adhesives developed from soy [68,69,105] and whey [58,59] proteins. Chemical crosslinking of soy protein isolate with glutaraldehyde was shown to increase the dry and wet lap shear strengths of plywood specimens made from cherry wood veneers by 31.5% and 115%, respectively [105]. Crosslinking of soy protein isolate with PAE resin also substantially improved the lap shear strengths of plywood specimens [68,69]. The wood composites bonded using soy protein-PAE resin adhesive had higher dry shear strengths than the commercial PF resin [68]. On the other hand, crosslinking of whey protein with polymeric methylene diphenyl diisocyanate enhanced the adhesive strength and water resistance of whey protein-based adhesives to such an extent that the whey protein-based adhesives demonstrated comparable performance to that of commercial aqueous polymer-isocyanate (API) adhesives [58,59].

**Key points**

- Chemical crosslinking of protein/peptides is a key step in improving the adhesive performance of formulations developed from hydrolyzed protein recovered from slaughterhouse waste.
- The amount of crosslinking agent in the formulation must be sufficient to effectively crosslink the peptides through reactions from amine and/or carboxyl groups.
- Bi- or multifunctional compounds possessing functional groups such as aldehyde (e.g., glutaraldehyde), isocyanate (e.g., 4,4-diphenylmethane diisocyanate), azetidinium (e.g., polyamideamine epichlorohydrin (PAE) resin), and epoxy group (e.g., triglycidyl amine) have shown great promise as crosslinking agents for peptides, and have resulted in adhesive formulations that satisfy the strength requirements of ASTM for urea formaldehyde resin adhesives, as well as the those of the China National Standard for type II plywood.

## 7. Conclusions and Outlook

From one-third to one-half of the live weight of animals processed by slaughterhouses is converted to a byproduct stream that could be an incredible resource of industrial protein that could be utilized in various value-added applications. However, there are still significant hurdles for deployment of this valuable protein resource as a feedstock for the production of bio-based products mainly due to: (i) the non-homogeneous nature of the feedstock and the limited solubility of its protein content; (ii) a negative perception associated with certain tissues such as SRM and/or non-edible offal; and (iii) limited resistance of protein-based products against water and microorganisms. This review summarizes the current technologies for protein extraction and recovery from slaughterhouse waste, and recent advances on utilizing such protein for development of wood adhesives. Hydrolysis of non-homogeneous proteinaceous feedstock has emerged as an essential step for solubilization and recovery of protein and/or peptides. The recovered protein hydrolyzates have shown good solubility and uniformity that imparts processability and applicability for functional applications. Of the various hydrolytic methods, protein hydrolysis using subcritical water appears to be a promising method as it excludes the use of chemicals and enzymes, and also allows some control of the molecular size of the hydrolysis products through the control of hydrolysis conditions. The hydrolyzed peptides show moderate adhesive strength and limited water resistance when used as a wood adhesive, but through chemical crosslinking of peptides, blending peptides with phenol-formaldehyde resin or latex-based resins, and curing under optimal conditions, peptides-based adhesive formulations have been produced that display comparable performance to that of phenol-formaldehyde resins that currently dominate the adhesive market. We believe that developing specific modification and/or crosslinking strategies through the analysis of available functional groups, and structure-property evaluation of colloidal aggregates of protein adhesive formulations will help in designing and development of superior performing adhesive systems. The current global focus to establish sustainable technology platforms to produce value-added products from renewable resources, coupled with the anticipated increases in global meat consumption offer many exciting opportunities where the protein recovered from slaughterhouses can be exploited as an invaluable source of industrial protein, particularly for the wood-based adhesives sector.

## Figures and Tables

**Table 1:** Protein concentration and yield of proteinaceous products recovered from hydrolyzate by different methods. Table adopted with copyright permission from Elsevier (for Mekonnen et al., 2013).

Recovery Methods	Thermal Hydrolyzate		Alkaline Hydrolyzate	
	Protein Concentration (%)	Yield (%)	Protein Concentration (%)	Yield (%)
Salt extraction	70.59 ± 2.56 ^a^	38.6	54.34 ± 3.38 ^a^	25.0
Salt extraction and ultrafiltration	83.04 ± 1.95 ^b^	33.0	71.68 ± 1.74 ^b^	22.0
Acid extraction	77.24 ± 1.46 ^b^	35.1	-	-
Water extraction	91.04 ± 1.73 ^c^	42.1	67.41 ± 0.76 ^c^	27.6

Values are reported as the mean ± standard deviation (*n* = 3). Means with the same superscript letters within a column are not significantly different at the 95% confidence level.
